# Obsessions Across Two Cultures: A Comparison of Belgian and Turkish Non-clinical Samples

**DOI:** 10.3389/fpsyg.2019.00657

**Published:** 2019-03-26

**Authors:** Fulya Ozcanli, Eva Ceulemans, Dirk Hermans, Laurence Claes, Batja Mesquita

**Affiliations:** ^1^Center for Social and Cultural Psychology, Faculty of Psychology and Educational Sciences, KU Leuven, Leuven, Belgium; ^2^Research Group of Quantitative Psychology and Individual Differences, Faculty of Psychology and Educational Sciences, KU Leuven, Leuven, Belgium; ^3^Centre for the Psychology of Learning and Experimental Psychopathology, Faculty of Psychology and Educational Sciences, KU Leuven, Leuven, Belgium; ^4^Research Group of Clinical Psychology, Faculty of Psychology and Educational Sciences, KU Leuven, Leuven, Belgium; ^5^Faculty of Medicine and Health Sciences, University of Antwerp, Antwerp, Belgium

**Keywords:** Obsessive-Compulsive Disorder, obsessions, dimensions, culture, cross-cultural differences

## Abstract

There is a growing interest in the role of culture in Obsessive-Compulsive Disorder, yet cultural studies to date have suffered from methodological limitations and lack a clear theoretical framework. In the current study, we adopted a rigorous methodological approach, and a clear cultural psychological framework. We compared the structure and frequency of obsessions in non-clinical samples (*N* = 706) from Belgium, a Western culture, and Turkey, a non-Western cultural context. Obsessions were measured by a newly compiled instrument that included a broad range of obsessions. Cross-cultural equivalence of the structure of obsessions was assessed both in the pooled data, and in each culture separately. At an abstract level, we found a two-factor structure that was cross-culturally invariant, and that fit both cultures equally well. These two types of obsessions each corresponded with a different model of agency. Compared to the Turkish sample, the Belgian sample reported more obsessions that can be understood from a disjoint (independent) model of agency as frequently found in Western cultures, whereas the Turkish sample, compared to their Belgian counterparts, reported more obsessions that can be tied to a conjoint (shared) model of agency as frequently found in non-Western cultural contexts. Differences in the prevalent types of obsessions were systematic and interpretable, therefore. In addition to the cross-culturally equivalent two-factor structure, we found culture-specific factor solutions; these solutions point to cultural differences in the experience of obsessions that have yet to be fully understood. In the Discussion, we outline future directions of the research on culture and obsessions.

## Introduction

Obsessive-Compulsive Disorder (OCD) is a heterogeneous condition that has been found across the world (Staley and Wand, 1995; Sasson et al., 1997). Obsessions, one of the two pillars of OCD, are intrusive and recurrent thoughts, ideas, images, and impulses. Individuals who experience obsessions often show compulsive behaviors, in an attempt to reduce anxiety related to obsessions (American Psychiatric Association, 2013). Although obsessions have been found in individuals around the world, it is not clear whether their experience is universal.

Research on OCD in non-Western samples has yielded both similarities and differences with obsessions in Western samples. Yet firm conclusions are hard to draw, because the existing research has examined cultural differences in the level of obsessions, without ascertaining that the dimensional structure of obsessions (i.e., the meanings and associations between obsessions) is the same across cultures. Moreover, no research before has started from a theory of why there might be cultural differences in obsessions. If cultural differences emerged in studies with non-Western samples, they often remained either unexplained or they were explained *post hoc*. A theoretical framework to understand why differences in obsessions might occur has been missing.

In the current study, we examine the dimensional structure of obsessions first, before establishing cultural differences in levels. We also sought to *understand* any cultural differences in obsessions, either in the dimensional structure or in their frequency. To the latter end, we started from the observation that obsessions pertain to the many ways in which actions lead to bad states of the world. We explore the possibility that cultural differences obsessions can be understood from cultural differences in the models of agency.

The study consists of an online questionnaire on obsessions, administered to non-clinical samples from two cultural contexts that seem marked by different models of agency, Belgium and Turkey.

### Obsessions

Much of the literature on OCD is devoted to establishing its dimensional structure (Radomsky and Taylor, 2005). The underlying idea is that different dimensions correspond to different types of OCD, with the possibility that these types would benefit from different treatment interventions (McKay et al., 2004). Not all research has been conducted with clinical samples. In fact, several Western studies have yielded evidence for the continuity between intrusive thoughts in non-clinical samples and obsessions in clinical samples (e.g., Van Oppen et al., 1995; Jonsdottir and Smari, 2000; Abramowitz et al., 2010). Below, we first synthesize the research on the OCD dimensions, as yielded by studies in Western contexts; we combine the results from both clinical and non-clinical samples.

#### Dimensions of Obsessions With Western Samples

**FIGURE 1:**
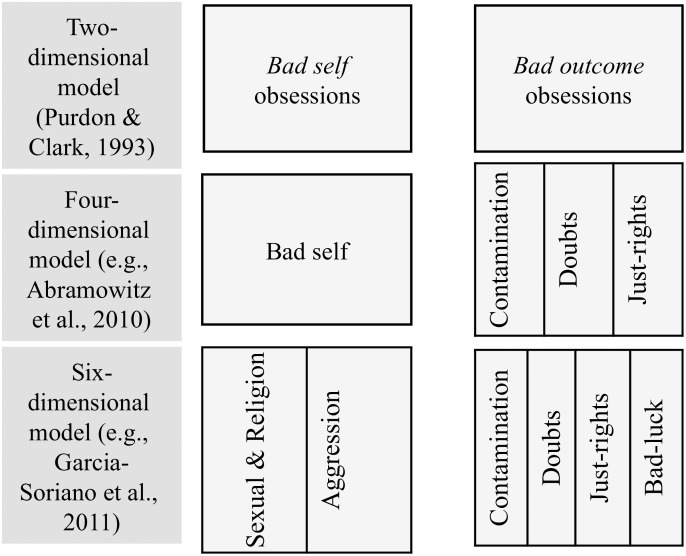
Different factor models of obsessions mapping onto a two-dimensional model.

Not all research with Western samples has yielded the same dimensions of obsessions. Depending on scales used, and preferred level of abstraction, researchers have found anywhere between one and seven dimensions (for a review, see McKay et al., 2004; Overduin and Furnham, 2012). Yet, the best-known symptom scales^1^ yielded four dimensions, each corresponding with a different theme^2^ (Abramowitz et al., 2003; Abramowitz et al., 2010) (see second level dimensions in Figure 1):

(a) *Bad-self obsessions*; e.g., attacking defenseless people, sexually assaulting strangers, shouting out blasphemous words in church.
(b) *Contamination obsessions*; e.g., catching a disease from public restrooms; harming others by spreading germs.
(c) *Doubt obsessions*; e.g., causing harm to loved ones unwittingly; the house burning down.
(d) *Just-right obsessions*; e.g., expressing oneself in the right way; everything in their right place.

There is some evidence that these dimensions can be grouped at a higher level of abstraction into a two-dimensional structure (see first-level dimensions in Figure 1). For instance, Purdon and Clark (1993), using the Revised Obsessional Intrusions Inventory (ROII), found only one dimension of obsessions in a sample of men and two dimensions of obsessions in a sample of women. For both men and women alike, they found a dimension combining aggressive and sexual obsessions that could be named as *bad-self* obsessions. For women only, they also found a dimension of obsessions referring to *bad-outcomes*; the latter dimension grouped items belonging to three of the four dimensions listed above (contamination, doubt, just right). The two dimensions, one reflecting *bad-self* and the other *bad-outcomes* were replicated for both sexes in many subsequent studies (Lee and Kwon, 2003; Belloch et al., 2004; Moulding et al., 2007). Therefore, these studies suggest a two-dimensional instead of the four-dimensional structure of obsessions that was introduced before.

There is some first suggestion that the lower-level dimensions can be mapped onto the higher-level dimensions of obsessions. One study, in which an obsessions scale was administered to participants of a large Spanish community sample provided evidence for this idea (García-Soriano et al., 2011). It showed a two factor as well as a six factor-solution (see Figure 1). The two factors corresponded to *bad-self* and *bad-outcomes* obsessions, respectively. The six factor solution was similar to the four-dimensional model described earlier (*bad-self*, contamination, doubts, “just-right” doubts), with the addition that *bad-self* obsessions were further split into sexual/religious, and aggressive obsessions. In sum, the Spanish study suggests that dimensionality of obsessions can be described at different levels of abstraction that each provide a fair characterization of the data, be it at a different level of detail.

Taken together, despite variations in the number of dimensions yielded by obsession research with Western samples, a coherent picture is emerging. Depending on the level of abstraction at which the dimensions were defined, two, four or six dimensions of obsessions emerged. Yet, each model distinguished between dimensions that describe individual agency, and dimensions that primarily focus on the (social) outcomes of events. We have labeled these dimensions *bad-self* and *bad-outcomes* obsessions.

#### Dimensions of Obsessions With Non-western Samples

In the past two decades, researchers have started to study obsessions in non-Western contexts as well (e.g., Tek and Ulug, 2001; Kim et al., 2005; Besiroglu et al., 2007; Matsunaga et al., 2008; Saleem and Mahmood, 2010; Chasson et al., 2013; Asadi et al., 2016). Often, the conclusion from these studies is that the same dimensions found for Western samples, can also be established in non-Western cultural contexts. This conclusion is not (yet) warranted, though, because the methods used in these studies suffer from at least two shortcomings.

The first shortcoming of studies conducted in non-Western cultural contexts is that they lack a careful examination of cross-cultural similarity in the meaning of obsessions. In many cases, Western scales have been imported, without checking if the meaning of the items (e.g., as inferred from the associations between different items) was similar in the given non-Western context. For instance, the Yale-Brown Obsessive Compulsive Scale (Y-BOCS; Goodman et al., 1989) was used in several studies with non-Western samples (e.g., Tek and Ulug, 2001; Saleem and Mahmood, 2010), but the researchers failed to check whether the same dimensions best described the data from the non-Western samples. They used the original Western labels for dimensions (e.g., just right doubts), without confirming whether these referred to similar obsessions (i.e., items) across different cultural contexts. In one study with Turkish participants (Tek and Ulug, 2001), the first dimension of obsessions was labeled “just right doubts,” implying a match with the similarly named dimension from Western research labeled. However, on closer examination, the “just right doubts” in the Turkish sample did not only consist of the “just-right doubts” found in Western samples (i.e., symmetry obsessions, ordering compulsions), but also of somatic obsessions (e.g., concern with illness). Researchers thus implicitly claimed similarity in dimensions without testing the “structural equivalence” (Van de Vijver and Leung, 1997) of the obsession items and their associations.

Was the dimension of “just right doubts” similar or different from the similarly named dimension in Western samples? The answer is that, without a Western comparison group, this question cannot be answered. In sum, non-Western research yielding dimensions that are merely reminiscent of the Western dimensions cannot be interpreted as evidence for cross-cultural similarity, without firmly *establishing* that the scale items have similar meanings (i.e., associations) across cultures. This can only be done in cross-cultural research.

Research that *does* include both Western and non-Western samples suffers from a second, related shortcoming. It compares the level of obsessions across different cultures, but without examining whether the dimensional structure is the same across them. Differences in frequency or significance are therefore compared, without establishing that the construct itself is cross-culturally similar in meaning. For example, a large-scale study among non-clinical participants from 16 different cultural sites concluded that the significant obsessions differed by culture (Radomsky et al., 2014). In this case, the researchers claimed that contamination intrusions were more distressing in Ankara (Turkey) and Thessaloniki (Greece) than in any other site; aggressive intrusions were more distressing in Chambéry (France) than in any other site. Interestingly, they based these claims on cross-national differences on the assumption that similar dimensions existed; however, without checking this assumption. The seven content areas of intrusions (i.e., contamination, aggression, doubt, religious, sexual, victimization, and “other” intrusions) were defined by International Intrusive Thoughts Interview Schedule (IITIS; Research Consortium on Intrusive Fear [RCIF], 2007), but a similar understanding of these content areas was assumed, rather than *established*.

In sum, most studies within non-Western samples, failed to test whether the dimensional structure of obsessions is cross-culturally similar. Both the lack of comparison groups and the failure to establish structural equivalence make it impossible to decide whether cultural similarities (and differences) in prevalence are meaningful and significant. It is impossible, therefore, to draw any firm conclusions on cross-cultural similarity in the experience of obsessions.

Rare exceptions of research that did attempt to establish the structural equivalence of dimensions of obsessions are not reassuring. Two studies including different cultural (ethnic) groups in the United States, found that that the theorized dimensions of OCD could be replicated for European American, but not African American samples (Garnaat and Norton, 2010; Williams et al., 2013). The first study used the Y-BOCS, and failed to establish structural equivalence with regard to the dimensions of obsessions between the Black and White American clinical participants (Garnaat and Norton, 2010). The second study used the Y-BOCS in an African-American clinical sample, and similarly failed to replicate the dimensions previously established for white clinical samples (Williams et al., 2013). In each case, confirmatory factor analysis yielded poor fit. Therefore, the few studies that used more rigorous statistical methods (e.g., confirmatory factor analysis) failed to replicate, even for African American samples, the structure obtained for Whites. Taken together, these results suggest the possibility of cultural differences in the dimensional structure of OCD, contrary to the general assumption of cultural similarity.

Going onward, we need cross-cultural studies that establish rather than assume cross-cultural similarity in the structure of OCD dimensions. Only based on cross-culturally meaningful dimensions, can we compare the experience of obsessions in different cultures. Therefore, in the current study, we will first investigate the structure of obsessions in two different cultures before comparing the cross-cultural prevalence of different dimensions of obsessions. Cultural comparisons can only be made for obsessions that are similar in meaning across cultures.

### A Cultural Psychology Approach to Obsessions

In this study, we start from a cultural theoretical framework. We suggest that important cultural differences in obsessions can be understood from differences in the culturally prevalent models of agency. Models of agency “are defined as implicit frameworks of ideas and practices about “how to be” that construct the actions of the self, of others, and the relationships among those actions. “They are typically invisible to those that engage or enact them.” (Markus and Kitayama, 2003, p. 6). “Disjoint” (independent) agency and “conjoint” (shared) agency have been distinguished as two prototypical models of agency that help theoretical analysis. Although both models of agency appear to occur universally, cultures differ with respect to the predominant model of agency.

A disjoint (individual) model of agency is foregrounded in many Western contexts. In these contexts, the independent self is seen as a source of agency. Actions are seen to be primarily the result of self-direction; that is, as resulting from an individual’s own desires, goals, intentions, or choices. Agency is constructed as contained or bounded within the individual, and actions are seen as a form of self-expression. A disjoint model of agency pays less attention to the role that others and the environment may have in the realization of certain outcomes. It comes with an “inside-out” perspective of the world: the perspective of an individual is central.

A “conjoint” (shared) model of agency is salient in many non-Western societies. According to this model, actions “do not come securely attached to individual agents” (Markus and Kitayama, 2003, p. 9); they do not result from the determination or intention of an individual alone. Rather, actions are necessarily achieved in interaction with socially important others and institutions, and in connection with others and the world, all of which (others, institutions, world) are focal and necessary for action. Agency is thus seen as an expression of individuals as *interdependent* (or connected) with their contexts, in which the individual is always referencing the environment, and also subject to its influence. Conjoint models of agency come with an “outside-in” perspective on the world: the consequences of actions to others, the relationship, and the social and world order are central contents.

We propose that *bad-self* obsessions correspond with disjoint models of agency, and that *bad-outcome* obsessions correspond with conjoint models of agency. Consistent with a disjoint model of agency, *bad-self* obsessions are threatening because they are considered diagnostic of the individual’s bad, irresponsible, and impure desires, goals, intentions, or choices, and are thus ultimately a reflection of the immoral core of the individual. Many (Western) OCD-researchers have framed obsessions in terms of a disjoint model of agency (Guidano and Liotti, 1983; Rachman, 2003; Ferrier and Brewin, 2005; Bhar and Kyrios, 2007), describing them as pertaining to the many ways in which the (imagined) acts of an individual can be bad, irresponsible, and impure, and thus calling them “ego-dystonic” (Clark, 2004). Consistent with a conjoint model of agency, *bad-outcome* obsessions focus on the interaction between an individual and their environment, and focus on the consequences of this interaction for everyone around (self as well as others) and for both the social and the divine order.

As the different models of agency occur cross-culturally, we expect both types of obsessions to be recognized across cultures. In support of this idea, previous research in Western contexts has established two-factor models of obsessions that correspond with *bad-self* and *bad-outcome* obsessions. Furthermore, cultural differences in the predominant model of agency lead us to expect cultural differences in the distribution of obsessions, with a higher prevalence of *bad-self* obsessions in Western cultures that foreground a disjoint model of agency, and a higher prevalence of *bad-outcome* obsessions in non-Western cultural context that gravitate toward conjoint models of agency (Weisz et al., 1984; Markus and Kitayama, 2003).

The current study seeks to explore to what extent cultural differences in obsessions can be understood from these differences in agency. To this end, we compare samples from a Western and a non-Western cultural context.

### The Current Study

In this study, we compared non-clinical samples from Flemish Belgium and from the Istanbul area of Turkey as examples of cultural contexts that foreground disjoint and conjoint models of agency, respectively. In previous research, Belgian samples have been found to endorse individualism and Turkish samples collectivism on the value dimension of Individualism (Hofstede, 2001). Similarly, in studies that specifically compared Belgian and Turkish samples, Belgian self-construals were more “autonomous” and less “related” than Turkish self-construals (Güngör and Phalet, 2011; Coskan et al., 2016). Finally, in a new self-construal scale with seven dimensions, Belgian samples were higher on the dimensions of self-containment (vs. connection to others), self-direction (vs. reception to influence), and self-expression (vs. harmony) than Turkish samples, for whom the reverse was true (Vignoles et al., 2016).

Our first goal was to examine to what extent the meaning structure of obsessions was similar for across cultures. We went beyond, and improved, even the common practice of establishing meaning equivalence by using statistical modeling that gave equal weight to both cultures. Rather than starting from the structure of Western (i.e., Belgian) experience, and checking if the non-Western (i.e., Turkish) data fit this structure (as for instance, a confirmatory factor analysis would do), we established models that maximally fit both the Western and the non-Western experience. In doing so, we investigated rather than assumed the extent of meaning equivalence of the dimensions of obsessions. Given that previous research found both cross-cultural similarities and differences in the structure of obsessions, we did not have a clear expectation about the degree to which the dimensions of obsessions would be universal. We expected any similarity to occur at the highest level of abstraction, and differences for dimensions at lower levels of abstraction.

Our second goal was to compare cross-cultural frequencies of obsessions, but only for those dimensions that were equivalent across cultures. We expected that any cultural differences in these frequencies could be meaningfully framed by cultural differences in the models of agency.

## Materials and Methods

### Participant Characteristics

The participants of the study were both undergraduate students and community individuals from Turkey and Belgium. The students were recruited from a moderately sized private university in Istanbul, Turkey, and from a large public university in Leuven, Belgium. They received course credit for their participation in the study. In both cultures, community samples were recruited through advertisements on social media, and in Belgium, through an adult vocational school. Community participants did not received any compensation, but upon completion of the questionnaire, they did enter a lottery that gave them a good chance of winning a 20 euro gift card.

The total number of participants was 706. We excluded twenty-five subjects who reported their country of birth outside of the target countries as well as two subjects who were outliers. Outliers were defined using approximate quartiles approach (Hoaglin et al., 1986).

Consistent with literature on country-level religiosity (e.g., Gebauer et al., 2012), we find that our Turkish sample is more religious than our Belgian sample. Religiosity was measured on a five-point scale that assessed the frequency of attending a place of worship, praying, reading a religious text (Bible, Koran), donation or time volunteered, and importance of religion in guiding the decisions and behaviors (Inozu et al., 2012). The main religious affiliations in the Turkish and in the Belgian samples were Islam and Christianity (Catholic), respectively.

The final samples in Turkey and Belgium did not differ with respect to mean age, gender composition. Small, but significant differences were found for education level. For an overview of the demographic characteristics of the final sample, see Table 1.

**Table 1:** Demographic characteristics.

	Turkey		Belgium			
	Community (*n* = 171)	Student (*n* = 158)	Community (*n* = 174)	Student (*n* = 177)	*X*^2^	*p*
Mean age	35.36	20.91	37.16	21.30	–	NS
(SD)	(9.51)	(1.36)	(13.31)	(1.33)	
Gender	112	139	136	150	2.91	NS
(female)	(72%)	(80%)	(78%)	(83%)	
Education	129		133		6.65	0.01
(university)	(86%)		(75%)		
Religion	249		155		–	–
(Muslim/Catholic)	(75%)		(44%)		
Mean	2.83		1.36			0.001
religiosity (SD)	(1.15)		(0.51)		


*The statistical significance for the age, gender composition, religious affiliation, and religiosity were calculated in the total samples (students and community participants together) across the countries.*

### Measures

#### Leuven Obsessional Intrusions Inventory (LOII)

To establish the dimensions of obsessions across two cultures, we needed an instrument that consisted of obsession items only, and that covered a wide variety of obsessions. Most existing instruments have measured a combination of obsessions and compulsions (e.g., OCI-R; Foa et al., 2002; PI-R; Van Oppen et al., 1995). The only known scale that exclusively focused on obsessions at the time we designed this research was the Revised Obsessional Intrusions Inventory^3^ (ROII, Purdon and Clark, 1994), which did not represent the full range of obsessions: religious obsessions, doubts, and just-right doubts were lacking. For the success of our mission, it was essential to include as many different obsessions as possible. Therefore, we decided to develop the *LOII* which, like the ROII, contained obsessive intrusion items only, yet unlike the ROII, sampled the broadest possible range of obsessional intrusions. Items were inspired by the ROII, but also on other commonly used OCD scales (see Footnote 1), including scales that were known to be in use in the Turkish context (but not developed in Turkey^4^; e.g., OCI-R; PI-R). Furthermore, we developed some new items (on doubts, just right doubts and religious obsessions) based on the detailed descriptions from research and clinical accounts (e.g., Rachman, 1997, 2003; Summerfeldt, 2007). In compiling the items for the LOII, we made sure to include all the different content areas found in the most common OCD-scales, until we reached saturation. Similar items from different scales were merged. All items of the LOII described obsessions; any items that in the original scales were formulated in terms of behaviors (e.g., I repeatedly check that my doors or windows are locked) were rephrased as a related obsession (e.g., Doubts about leaving a door or window unlocked). The final scale consisted of 50 items (see Table 2, for a full list).

**Table 2:** Two factor model of obsessions: Results of EFA based on the rotated factor loadings.

Factor 1: Bad-self obsessions	Combined	R-TR	R-BE
Thoughts, images or impulses of harming myself or others	0.60	0.63	0.55
Unwanted thoughts, images or impulses contradictory to my sexual orientation	0.60	0.70	0.40
Sexually unwanted thoughts, images or impulses involving people with whom sex is inappropriate	0.60	0.63	0.52
Thoughts of acting immorally	0.60	0.64	0.50
Thoughts, images or impulses to push someone (from the bridge, out of the window, into a running traffic…)	0.59	0.62	0.56
An impulse to blurt out obscenities in public	0.58	0.61	0.53
Sexually unwanted thoughts, images or impulses involving strangers	0.58	0.61	0.51
Sexually unwanted thoughts, images or impulses contradictory to my moral values	0.57	0.57	0.56
Thoughts, images or impulses about attacking someone	0.57	0.52	0.67
A thought, image or an impulse to publicly expose myself	0.56	0.59	0.51
Unwanted thoughts, images or impulses involving violent sexual acts	0.55	0.55	0.55
Thoughts, images or impulses to hurt defenseless people	0.55	0.54	0.61
Thoughts, images or impulses involving weapons or sharp objects	0.54	0.53	0.54
An impulse to shout out blasphemous words	0.53	0.57	0.42
Sexually unwanted thoughts, images or impulses involving defenseless people	0.53	0.51	0.56
Thoughts, images or impulses to steal something	0.45	0.39	0.56
An impulse to do inappropriate things in a religious context	0.44	0.49	0.31
Thoughts, images or impulses to drive a car into something or someone	0.41	0.46	0.42
Inappropriate thoughts or images involving important religious figures (prophet, imam, priest…)	0.41	0.47	0.26
An impulse to swear in public	0.39	0.45	0.30
Thoughts, images or impulses to hurt animals	0.34	0.31	0.46
Doubts about my religious faith	0.31	0.42	0.01
**Factor 2: Bad-outcome obsessions**			
Doubts or images about being contaminated by germs	0.72	0.72	0.71
Doubts or images of contamination after touching garbage or garbage bins	0.68	0.69	0.66
Doubts or images of contamination by touching publicly used door knobs	0.63	0.61	0.69
Doubts or images about catching a disease from public restrooms	0.63	0.65	0.57
Doubts or images about being contaminated, even after slight contact with bodily fluids (sweat, saliva, urine…)	0.63	0.65	0.57
Doubts about being contaminated without knowing it	0.60	0.58	0.63
Doubts or images of contamination after touching an animal	0.58	0.58	0.57
Doubts or images about my hands being dirty after touching money	0.54	0.52	0.57
Doubts about catching a fatal disease (AIDS, Ebola …)	0.53	0.55	0.51
Doubts about whether I switched off the lights, stove or iron	0.51	0.55	0.46
Doubts about causing disastrous consequences to loved ones or myself by my being reckless	0.50	0.57	0.38
After having talked to someone, doubts about whether I expressed myself in the right way	0.49	0.51	0.46
After completing a task, doubts about whether I did things in the way they were supposed to be done	0.48	0.49	0.48
Doubts about being poisoned by chemical substances (household cleaning products, poisonous substances, radiation…)	0.46	0.53	0.30
Doubts about leaving a door or window unlocked	0.44	0.47	0.41
After having done things, doubts about whether I actually carried them out	0.43	0.44	0.43
Doubts that my words or acts will be interpreted as hurtful	0.43	0.44	0.43
Doubts that objects might be arranged in a wrong way	0.43	0.49	0.30
Doubts about my forgetfulness that will put people around me at risk	0.42	0.41	0.45
Doubts about having an illness of which the existence is not yet known	0.42	0.42	0.42
Thoughts or images about accidents involving a loved one	0.42	0.42	0.41
After being done with a project, doubts of whether my work is still being incomplete	0.40	0.40	0.41
Doubts about skipping important information while reading a book, newspaper or magazine	0.40	0.47	0.29
Doubts about accidentally causing harm to other people without knowing it	0.40	0.42	0.36
Doubts about harming others by spreading germs	0.39	0.39	0.38
Doubts about accidentally hitting a pedestrian while driving	0.38	0.41	0.34
Doubts that I might offend God	0.36	0.45	0.02
Doubts about performing a religious task or ritual in the right way	0.34	0.41	0.13


*“Combined” refers to factor loadings of the pooled data. “R-TR” and “R-BE” refers to the rotated factor loadings of Turkey and Belgium, respectively, toward the combined sample solution.*

Items were presented with the following instruction: “Below you will read descriptions of certain thoughts, images, or impulses that many people experience in their daily lives. These thoughts pop up involuntarily. Please, read each statement carefully and answer how frequently these thoughts come to your mind.” The frequency of each thought was rated on a five point scale from 0 “never” to 4 “very often.”

We developed the initial pool of items in English, and translated the scale into Dutch and Turkish, respectively. In addition, we back-translated the Dutch version into the Turkish version to establish meaning equivalence between the Turkish and Dutch versions (Beaton et al., 2000). Differences between translations were resolved through discussions between the translators.

### Procedure

The participants were asked to first read the consent form, which informed them that they were free to either omit questions or withdraw from the study altogether, without experiencing any disadvantage. Participants then completed a demographic questionnaire that assessed their age, sex, educational status, and country of birth. Finally, they rated the frequency with which they experienced each obsession. The order of presentation of the obsession items was randomized.

We used an online platform for the data collection (Qualtrics, Provo, UT, United States) as a way both to access community samples and to protect the anonymity of the participants. We reasoned that anonymity was necessary given the sensitive content of many of the items. Previous research has shown that online data collection reduces the tendency toward socially desirable answers (Bargh et al., 2002; Henderson et al., 2012), which we feared would have prevented us from gaining insight into the actual frequency of obsessions, especially those on aggression, sexuality, and religion.

## Results

### Analysis Strategy

Below, we present the data in three sections. The first section presents the dimensions of obsessions that the Turkish and Belgian samples had in common based on pooled data. In the second section, we present the culturally unique dimensions that best explained the data in each culture (by conducting separate analysis in each culture). Finally, in the third section, we examined the cultural variations in the frequency of obsessions based on the common structure (based on pooled data).

To analyze the underlying structures of obsessions, we used exploratory factor analysis (EFA) for both the pooled data and the data from each cultural group separately. For ease of interpretation, we chose varimax rotation (Fischer and Fontaine, 2010). Before the analysis, we centered each item based on cultural group mean scores to prevent EFA to be affected by mean differences (Leung and Bond, 1989; Fischer, 2004). The decision on the number of factors to be retained was based on three different considerations. An examination of (a) scree test (Cattell, 1966), (b) parallel analysis (Horn, 1965), and (c) interpretability of the dimensions. Parallel analysis is often recommended as the best method to estimate the true number of factors (Lance et al., 2006), especially for the cases in which raw data are not normally distributed (O’Connor, 2000), which was the case for our data.

In order to establish whether the factors were cross-culturally invariant, we used Procrustean rotations (Van de Vijver and Leung, 1997), rotating each culture’s loadings onto the loadings that emerged from the factor analysis of the pooled data and onto the loadings that emerged from the factor analysis of the other culture’s data (see below). After target rotation had been carried out, factorial agreement was estimated, using Tucker’s coefficient of agreement (Tucker’s phi). As a rule of thumb, when Tucker’s coefficient values were 0.90 and higher, they indicate strong factorial similarity (Van de Vijver and Poortinga, 2002). Cronbach alpha coefficients are calculated to determine the internal consistencies of the scales (see Supplementary Table S1).

### Common Dimensions of Obsessions

Our first analyses sought to establish a model of OCD that would work in the Turkish as well as in the Belgian context. The scree test showed two elbows indicating a two-factor and a four-factor solution. In addition, parallel analysis offered five and six factor solutions. The two, four, five, and six-factor solutions were interpretable, as they matched similar solutions reported by previous research. However, further analyses showed that only the two-factor solution was fully congruent across the two cultural groups. When comparing the culture-level solutions with the pooled data solution, Tucker coefficient values showed factorial similarity between Turkey and Belgium for both factors in the two-factor solution. However, for solutions with more factors, Tucker coefficient values suggested differences for two or more of the factors (for example, see the six-factor solution in the Supplementary Table S2). Therefore, we pursued the two-factor solution.

The two-factor model was similar to two-dimensional models established in earlier research (see Purdon and Clark, 1993; Lee and Kwon, 2003). The two factors are shown in Table 2. The dimension of *bad-self* obsessions includes a mixture of what in previous OCD-literature has been called sexual, aggressive, and religious/blasphemous thoughts; the dimension of *bad-outcome* obsessions, in terms of the same OCD literature, consists of contamination obsessions and a mixture of doubts (including just-right doubts). Supplementary Table S1 presents the Tucker’s Phi indexes and internal consistencies of the dimensions. A closer look at the dimensions in terms of the models of agency suggests that *bad-self* obsessions all describe individual acts that are somehow indicative of the bad or immoral core of the self. In contrast, *bad-outcome* obsessions, though at times the result of individual agency, are no expression of a low moral caliber of the individual; rather, these obsessions refer to involvement in a bad, impure, or immoral world.

### Country-Specific Dimensions of Obsessions

Our second set of analyses sought to establish, for each culture separately, the dimensions that best fit the data. The number of factors that emerged for the Turkish data was different from the number that emerged from the Belgian data (see below). To establish the degree of meaning equivalence, we therefore constrained the number of factors in the source country to the number of factors that had emerged for the target country. We conducted Procrustes rotations on these factors, thus maximally aligning the loadings for the source and the target country. We examined the values of the resulting Tucker’s coefficient as an index for cross-cultural equivalence.

#### The Turkish Model

**FIGURE 2:**
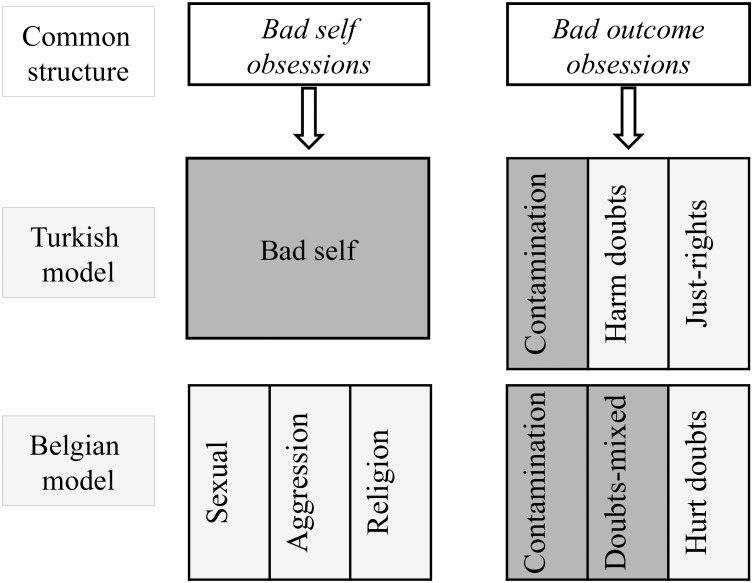
Fit between Turkish and Belgian models. The shaded areas show dimensions of factorial agreement, based on Tucker congruence values (>0.90). This means that the factor loadings in the other culture fit these dimensions well. Non-shaded areas describe dimensions that do not fit the factor loadings of the other culture.

A four-factor model fit the data of the Turkish sample best (see Supplementary Table S3, for the item loadings per factor and the rotated fit loadings of the Belgian sample). The first factor consisted of *bad-self* obsessions, including aggressive, sexual, religious thoughts (e.g., unwanted thoughts, images or impulses contradictory to my sexual orientation). The second factor included contamination obsessions (e.g., doubts or images of contamination after touching garbage or garbage bins) and some doubts. The third factor contained just-right doubts, including some religious concerns (e.g., after having talked to someone, doubts about whether I expressed myself in the right way). The fourth factor involved doubts about accidentally causing harm (e.g., Doubts about causing disastrous consequences either to loved ones or to myself by my being reckless). Despite the seeming similarity of this four-factor model to the four factors discussed in the “Introduction” (see, e.g., Abramowitz et al., 2010), factorial agreement across cultures could only be established for the first two factors, and not for the latter two. Supplementary Table S4 shows that Tucker’s coefficient values are good for *bad-self* and *contamination* obsessions, but not for *just-right* *doubts* and *accidental harm doubts* (for a graphical representation, see Figure 2).

#### The Belgian Model

A six-factor model best described the Belgian data (see Supplementary Table S5 for the item loadings per factor and the rotated fit loadings of Turkish sample). The first factor consisted of contamination obsessions (e.g., doubts or images about being contaminated by germs). The second factor contained “doubts” (e.g., doubts about whether I switched off the lights, stove or iron), and “just right” doubts (e.g., after having done things, doubts about whether I actually carried them out). The third and fourth factors consisted of *bad-self* obsessions involving aggression (e.g., thoughts, images or impulses to hurt defenseless people) and sexuality (e.g., sexually unwanted thoughts, images, or impulses involving strangers), respectively. The fifth factor consisted of doubts with themes of both “being hurt” and “hurting others” (e.g., doubts that my words or acts will be interpreted as hurtful). Like the third and the fourth factor, the sixth factor consisted of *bad-self* obsessions; this time with a theme of religion/blasphemy (e.g., an impulse to shout out blasphemous words). As shown in Supplementary Table S6, factorial agreement across cultures was established for the first two factors, but not for any of the other factors. Tucker’s coefficient values were only acceptable for contamination obsessions and doubts (for a graphical representation, see Figure 2).

### Cultural Differences in the Frequency of Obsessions

We assess cross-cultural differences in the relative frequency of the common dimensions of obsessions, *bad-self* and *bad-outcome* obsessions. Controlling for culturally unique response tendencies, we used culturally centered standardized scores of obsession, which are achieved by subtracting the respective cultural mean scores across all items from the individual item score (Bond, 1988; Leung and Bond, 1989). Because the data were not normally distributed according to Kolmogorov–Smirnov tests, we applied non-parametric Mann–Whitney *U*-tests to examine the significance of between country frequency differences.

**FIGURE 3:**
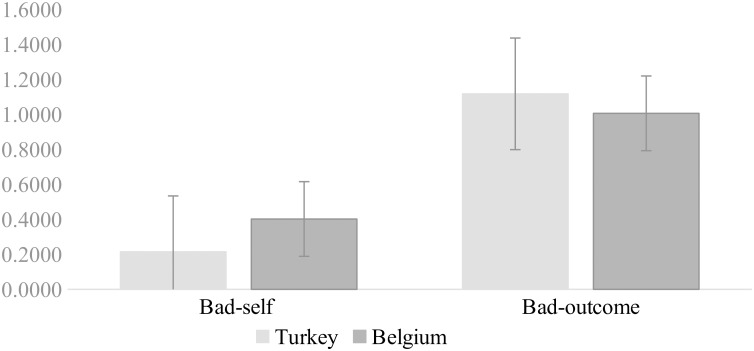
Relative frequencies of two-factor model of obsessions in Turkey and Belgium.

The analysis revealed cultural differences with respect to both *bad-self* obsessions and *bad-outcome* obsessions (see Figure 3). As expected, we found relatively more *bad-self* obsessions in Belgium compared to Turkey (*Mdn_Turkey_* = 0.21, *Mdn_Belgium_* = 0.40) (*U* = 77.41, *z* = 7.77, *p* < 0.001, *r* = 0.29), and relatively more *bad-outcome* obsessions in Turkey compared to Belgium (*Mdn_Turkey_* = 1.11, *Mdn_Belgium_* = 1.00) (*U* = 52.03, *z* = -2.16, *p* < 0.05, *r* = -0.08).

## Discussion

In spite of a growing body of evidence suggesting that even basic psychological processes may vary dramatically across cultures, the belief among mental health researchers that Western-based instruments capture non-Western experience remains almost unchallenged (but see, Kleinman, 1987; Bass et al., 2007). Cross-cultural research on OCD forms no exception to this belief. The last few decades have shown a growing interest in the role of culture in OCD. Yet, research on culture and obsessions is still in its infancy. The current research sought to make some first progress toward a cultural psychology of obsessions. To this end, we furthered the research both conceptually and methodologically.

Conceptual progress was made by adopting a cultural psychological perspective on obsessions. We started from the notion that obsessions are about (imagined) acts that have a threatening content, and postulated that cultural differences in the most prevalent obsessions could be understood from the cultural models of agency. Hence, we made meaningful and systematic predictions about the types of cultural differences in obsessions that were to be found across cultures. To our knowledge, we are among the first to adopt a theoretical, if exploratory, approach to cultural differences in OCD.

Methodological progress was made by establishing, rather than assuming, the degree of meaning equivalence in obsessions. Rather than assuming that the obsession dimensions established by Western research would apply in non-Western settings, we carefully compared the underlying dimensions of obsessions in both cultures. These analyses were in the first place exploratory. We first analyzed the structure of the pooled data (i.e., the data from both cultures together), and identified dimensions of obsessions that converged between cultures. We then examined the culturally unique structures, to do maximal justice to each culture separately.

Our Turkish and Belgian samples were selected on theoretical grounds: Prior research suggested that the salient model of agency differs between those cultural contexts. A disjoint model of agency, in which agency is constructed within the individual, is foregrounded in a Belgian cultural context; a conjoint model of agency, in which agency is constructed as tied to others and the environment, is salient in a Turkish cultural context. Sample selection on theoretical grounds increases the likelihood that cultural differences in obsessions are found, should they exist.

In the next sections, we discuss our findings: (1) the cross-culturally similar dimensions of obsessions, (2) the culture-specific dimensions, and (3) cultural differences in the frequencies of the culturally shared dimensions. The results suggest both cultural similarities and variations in obsessions.

### Cross-Culturally Shared Dimensions of Obsessions

We first established the culturally shared dimensions of obsessions. Obtaining similar factor structures in distinct populations, is considered proof that an instrument measures the same constructs in these populations (Lacasse et al., 2014). Hence, if our obsessions scale yields similar factorial structures across cultures, this could be taken as an indication of the “universality” of the underlying dimensions (Fischer and Fontaine, 2010, p. 180).

Our analyses revealed two broad dimensions of obsessions that could be described as *bad-self* and *bad-outcomes* obsessions, whose meaning was equivalent in both cultural contexts. These dimensions do not only replicate dimensions that have been found in earlier studies on obsessions (Purdon and Clark, 1993; Lee and Kwon, 2003), but they also can be understood to represent the respective cultural models of agency. The *bad-self* dimension that consists of a mixture of sexual, aggressive, and religious/blasphemous thoughts and impulses, pertains to acts that are the result of individual agency, and that can be seen as expressions of a bad, irresponsible, or impure individual. In other words, these acts seem threatening because they reveal that the agent is bad at core. The *bad-outcomes* dimension that is constituted by contamination obsessions and a mixture of doubts, pertains to acts that lead to a threatening state of the world. The outcomes of these acts are more defining of the obsessions than what they express about the individual. Often the agency in *bad-outcomes* obsessions results to an interaction between the individual and their environment. The act of touching a door knob only leads to contamination when the door knob is full of germs, my own acts or words are only threatening when interpreted by others as hurtful, and leaving the iron on makes the world a bad place when it leads to house to catch on fire. In all of these *bad-outcome* obsessions, the agency is an interaction with the context.

The two-dimensional structure of obsessions that we found for both cultures corresponds with different models of agency is also suggested by research by Lee and Kwon (2003). These researchers similarly found *bad-self* obsessions (to which they refer as to “autogenous obsessions”) and *bad-outcome* obsessions (referred to as “reactive obsessions”). In addition, they examined the associated appraisals and control reactions for each dimension of obsessions (Lee and Kwon, 2003; Seo and Kwon, 2013). Consistent with a disjoint model of agency, they found that *bad-self* obsessions were associated with an “importance of thought” appraisal (e.g., because I’ve had this thought, I must want it to happen), with “negative inferences about the self” (e.g., because I’ve had this thought, I am a bad person), and with the belief of having “control over thought” (e.g., because I’ve had this thought, I’m out of control). These associations do support an interpretation of *bad-self* obsessions as expressive of the intentions, desires, and indeed, character of the individual. Consistent with a conjoint model of agency, Lee and Kwon (2003) found an association between *bad-outcome* obsessions with feeling responsible for *preventing* the feared outcomes (e.g., because I’ve thought of bad things that might happen, I must act to prevent them). Again, this suggests that the outcomes are not necessarily due to individual agency, even if the individual is involved. It is the interaction of an individual with their environment that appears to be responsible for the outcomes.

The current study fails to confirm cross-cultural similarity in meaning at a more detailed level than the global binary level of *bad-self* versus *bad-outcomes*. Factor-analyses on the *pooled* data also yielded four-, five-, and six-factor solutions that could be meaningfully labeled. However, attempts to replicate these solutions for each culture separately were unsuccessful. This suggests that the meanings of the obsessions were similar at the abstract, binary level, but not at the more detailed level as captured by the other factor solutions. In conclusion, it is possible to cross-culturally compare the frequencies of the *bad-self* and *bad-outcome* obsessions; not the frequencies of the other dimensions.

### Culture-Specific Dimensions of Obsessions

Next, we established, for each culture separately, the dimensional structure of obsessions that best described the data; i.e., we separately modeled the Belgian and the Turkish data. Importantly, the culture-specific structures explained more variance in each cultural context than did the cross-culturally shared structure. This suggests that culture plays a role in the experience of obsessions. Furthermore, it was neither possible to fit the Turkish data into the Belgian model, nor to fit the Belgian data into the Turkish model. With few exceptions, the dimensions in the culture-specific structures were unique to either the Turkish or the Belgian context.

We make two observations about the differences. The first is that the Turkish sample sees just one type of *bad-self* obsessions, where the Belgian sample distinguishes three: In the Belgian sample, sexual, aggressive, and religious obsessions loaded onto different dimensions. It is possible that the cultural focus on actions originating from the individual leads to finer discriminations of such thoughts. This would be consistent with theorizing in psychological anthropology on “hypercognition,” a term referring to the more elaborate cognitive representations of concepts that are of cultural concern (Levy, 1984). If the Belgian group were to hypercognize *bad-self* obsessions, we would predict that they have a more elaborate narrative on *bad-self* obsessions and that *bad-self* obsessions would be more prevalent. This is consistent with the latter prediction (see below), but future research should bear out whether the narrative about *bad-self* obsessions are in fact more detailed in Belgian (and other Western) contexts. It should be noted that we do not find the mirror image for *bad-outcome* obsessions in the Turkish group: the Turkish sample did not make finer discriminations within the *bad-outcomes* obsessions than the Belgian sample.

The second observation pertains to religious obsessions, whose significance is quite different in the Belgian and the Turkish samples. Religious obsessions in the Belgian sample emerged as a separate dimension, whereas religious obsessions in the Turkish context seemed to occur both as *bad-self* obsessions, and described the person as sinful (e.g., shouting out blasphemous words), and as “just right doubts,” which seemed to be concerned with deviations from the “right” order of the universe (e.g., performing a religious task in the right way). The reason that the Turkish dimension of “just right doubts” did not correspond with the corresponding Belgian dimension (i.e., there was no “factorial agreement”) was in fact due to these religious obsessions (see Supplementary Tables S3, S5: the rotated loadings for the religious items were the lowest). A likely reason for these differences lies in the differences in religiosity between the samples. The low levels of religiosity make religious obsessions stand out in the Belgian sample; there is simply no variance. In the Turkish cultural context, religion is a significant part of life, thus might have been reflected in the structure of obsessions as well.

In conclusion, the dimensions of obsessions emerging from our data were, for the most part, different for the Belgian than for the Turkish sample. Some differences appear to be meaningful; for many others we lack as yet a guiding theory (see also the section “Limitations and Future Directions”). Comparison with previous research raises some questions as well. The dimensions of the Turkish unique structure seem to be similar to the four dimensions reported in previous Western research (Abramowitz et al., 2003). In the absence of cross-cultural samples in this earlier research, and given that our scale differs from the scale used there (the Y-BOCS), we cannot draw conclusions about the similarities or differences between earlier research and our Turkish sample. Instead, we can state with certainty that factorial invariance on two dimensions could *not* be established for the Turkish and the Belgian samples. It thus appears that dimensions of obsessions, while cross-culturally invariant at an abstract level, vary significantly once they are described at the more detailed level that captures the cultural experience best.

It is this detailed level that may be most relevant to the clinical practice. Not coincidentally, clinical studies commonly describe more specific symptom structures (for a review see McKay et al., 2004). From a cultural perspective, it is interesting to note that even if clinically relevant experiences are tied in with specific cultural meanings and practices, the dimensions of obsessions may also be captured at a more abstract, universal level.

### Cultural Differences in the Frequency of Obsessions

Our second objective was to investigate differences in the cultural frequencies of obsessions. Although other studies have documented obsessions in non-Western samples, inferring both similarities and differences with the obsessions in Western samples, almost none checked construct equivalence of the obsession scales that were used. This has made interpretation of cultural variation in frequencies of obsessions difficult. The current study established structural equivalence of obsessive dimensions prior to measuring cultural variations in endorsement. We are, therefore, confident that we compared similar constructs across cultures.

We established cross-cultural differences in the relative prevalence of *bad-self* and *bad-outcome* obsessions. *Bad-self* obsessions were relatively more prevalent in the Belgian sample compared to Turkish sample, and *bad-outcome* obsessions were relatively more prevalent in the Turkish sample compared to the Belgian sample. Given the larger emphasis on disjoint agency in the Belgian context, it is not surprising to find that Belgian participants more frequently had intrusions about acts for which they bore exclusive responsibility compared to their Turkish counterparts, especially when these acts reflected on their core preferences or desires. Given the larger emphasis on conjoint agency in the Turkish context, it is also not surprising to find that Turkish participants were more focused on the negative consequences of acts or events compared to their Belgian counterparts.

Of interest is also that, across cultures, *bad-outcome* obsessions were more prevalent than *bad-self* obsessions. This result is consistent with previous studies, both from Western and non-Western samples (e.g., Sasson et al., 1997; Radomsky et al., 2014).

### Limitations and Future Directions

Several features of this study limit our conclusions. First, although the items for the Leuven Obsession Inventory were broadly sampled, they were derived from existing scales. Even though these scales are also the ones currently in use in Turkey, they have been developed in Western cultural contexts. While our study thus advanced our insights into the culturally shared and non-shared obsession dimensions, and allowed us to compare and understand the relative prevalence of different obsessions across cultures, it is still possible that we missed out on obsessions that have escaped the attention of OCD researchers so far. Future research may want to start from an even broader range of items that are generated either by ethnographic (clinical) research, or informed by culturally sensitive theoretical models. For instance, given the focus on conjoint models of agency on the consequences of acts for the social order, we may add obsessions about violations of that order: not meeting social expectations or obligations, or not properly fulfilling one’s social roles. It is possible that we learn more about the culture-specific dimensions of obsessions, if we add a larger range of obsessions –should they occur.

Second, this study includes non-clinical samples only. Current models suggest that OCD is a dimensional disorder. This means that the endorsement levels but not the dimensional structure of the obsessions is different between non-clinical and clinical samples. Empirical evidence for this assumption is limited, though, and consists of studies in Western cultural contexts only. Moreover, the evidence so far has confirmed that the dimensional structure of obsessions found for clinical samples also fits the data for non-clinical samples (e.g., Van Oppen et al., 1995; Jonsdottir and Smari, 2000; Abramowitz et al., 2010), but there is no evidence for the reverse. Future studies should, therefore, examine whether there is continuity between non-clinical and clinical samples in both Western and non-Western cultures. A next step in our research would be to compare Belgian and Turkish clinical samples and examine whether the structure found in the current research can be replicated.

Third, though we included large sample sizes in this study, analyses were explorative. Future research should replicate the cultural differences in dimensions of obsession in Turkey and Belgium. Replication is all the more important, because one of the largest differences between Turkish and Belgian OCD-dimension was the degree of differentiation within the *bad-self* obsessions: Belgian participants made finer distinctions than the Turkish participants. This finding does not consistently resonate with previous research. For example, in a study by Tek and Ulug (2001), Turkish OCD patients distinguished between aggressive obsessions on the one hand, and sexual/religious obsessions, on the other. Future research should do confirmatory research with the two cultures to assess the stability of the dimensions obtained.

Fourth, the research compared two cultural groups, which were selected based on theoretical criteria. However, research needs to be expanded to other cultures that differ on relevant dimensions from the current two cultures, to ascertain whether the two-factor structure is in fact universal.

## Conclusion

Lemelson (2003) argued that “OCD symptoms can act like a lens that magnifies certain aspects of culture that have salience for individual experience.” Supporting this claim, the results of the present study suggested cultural differences with respect to both the meanings and the relative frequency of obsessions. We proposed that these cultural differences can be understood from differences in the culturally dominant perspectives on the agency. Consideration of cultural factors in the experience of obsessions may benefit research on the etiology of obsessions, and ultimately, on the treatment of obsessions.

## Ethics Statement

This study was carried out in accordance with the recommendations of the Social and Societal Ethics Committee (SMEC, University of Leuven). The protocol was approved by SMEC. We obtained consent from the participants via online consent forms.

## Author Contributions

FO, DH, and LC designed the questionnaire of the study. All authors contributed the general design of the study and approved the final manuscript. EC conducted the statistical analysis. FO and BM wrote the first draft of the manuscript.

## Conflict of Interest Statement

The authors declare that the research was conducted in the absence of any commercial or financial relationships that could be construed as a potential conflict of interest.

## Footnotes

1 The consulted symptom scales are: The Yale-Brown Obsessive Compulsive Scale (Y-BOCS; Goodman et al., 1989); Revised Obsessional Intrusions Inventory (ROII; Purdon and Clark, 1993, 1994); the Padua Inventory (PI; Sanavio, 1988), the Revised Padua Inventory (PI-WSUR; Burns et al., 1996); (PI-R; Van Oppen et al., 1995); the Obsessive Compulsive Inventory Revised (OCI-R; Foa et al., 2002), the Vancouver Obsessional Compulsive Inventory (VOCI; Thordarson et al., 2004); and the Dimensional Obsessive Compulsive Scale (DOCS; Abramowitz et al., 2010) (for a review, see McKay et al., 2004). These scales differ in the number of items, the types of items included (e.g., the ratio of compulsions to obsessions), and the question format (e.g., percentage of time spent on an intrusion vs. presence of intrusion).

2 These different dimensions have been given different names in the literature. For example, *bad-self* obsessions are also called as autogenous obsessions (Lee and Kwon, 2003; forbidden thoughts, Abramowitz et al., 2010, pure obsessions, Baer, 1994; and repugnant thoughts, Rachman, 1997). Doubt obsessions are sometimes called as pathological doubts (Rasmussen and Eisen, 1991).

3 At the time of the development of LOII, to our knowledge, ROII was the only intrusions scale that was widely used. However, after data collection, we learned about another intrusions scale that covers a wide range of obsessional intrusions (Inventario de Pensamientos Intrusos Obsesivos, INPIOS) (García-Soriano et al., 2011). We discuss the results from this scale in the Introduction (p. 3, 4).

4 The first author is a clinical psychologist from Turkey, and is not aware of any indigenous obsession scales.
