# Supplementary material for: Obsessions Across Two Cultures: A Comparison of Belgian and Turkish Non-clinical Samples
Source: Front Psychol. 2019 Mar 26;10:657. doi: 10.3389/fpsyg.2019.00657 (PMC6443853; doi:10.3389/fpsyg.2019.00657)
Supplement: Supplementary file 1 [file Data_Sheet_1.pdf]

Supplementary Material

Obsessions Across Two Cultures: A Comparison of Belgian and Turkish Non-Clinical Samples

Fulya Ozcanli,*, Eva Ceulemans, Dirk Hermans, Laurence Claes, and Batja Mesquita

Correspondence: Fulya Ozcanli: fulya.ozcanli@kuleuven.be

1 Supplementary table

Table 1. Two-factor model: Internal consistency coefficients and Tucker congruence values

	Bad-self obsessions		*Bad-outcome* obsessions	
	Turkey	Belgium	Turkey	Belgium
Cronbach Alpha	.92	.89	.90	.86
Tucker Phi	.99	.97	.98	.97

2 Supplementary table

Table 2. Six- factor common solution: Results of EFA based on the rotated factor loadings.

Factor 1: Doubts	Combined	R-TR	R-BE
After having talked to someone, doubts about whether I expressed myself in the right way	.65	.66	.60
Doubts that my words or acts will be interpreted as hurtful	.61	.60	.62
After having done things, doubts about whether I actually carried them out	.60	.58	.60
After being done with a project, doubts of whether my work is still being incomplete	.58	.53	.61
Doubts about whether I switched off the lights, stove or iron	.53	.51	.53
After completing a task, doubts about whether I did things in the way they were supposed to be done	.53	.51	.51
Doubts about causing disastrous consequences to loved ones or myself by my being reckless	.49	.56	.40
Doubts about accidentally causing harm to other people without knowing it	.48	.47	.52
Doubts about my forgetfulness that will put people around me at risk	.47	.42	.51
Doubts about accidentally hitting a pedestrian while driving	.41	.45	.37

Thoughts or images about accidents involving a loved one	.38	.32	.45
Doubts about leaving a door or window unlocked	.37	.28	.45
Doubts about skipping important information while reading a book, newspaper or magazine	.37	.42	.26
Doubts that objects might be arranged in a wrong way	.35	.37	.36
Factor 2: Sexual & Blasphemous			
Sexually unwanted thoughts, images or impulses contradictory to my moral values	.67	.68	.63
Sexually unwanted thoughts, images or impulses involving people with whom sex is inappropriate	.57	.62	.38
Unwanted thoughts, images or impulses contradictory to my sexual orientation	.56	.66	.33
Sexually unwanted thoughts, images or impulses involving strangers	.53	.56	.38
An impulse to blurt out obscenities in public	.52	.55	.55
An impulse to shout out blasphemous words	.51	.53	.44
Unwanted thoughts, images or impulses involving violent sexual acts	.51	.50	.54
An impulse to do inappropriate things in a religious context	.48	.48	.28
Inappropriate thoughts or images involving important religious figures (prophet, imam, priest…)	.47	.49	.34
Thoughts of acting immorally	.46	.54	.33
A thought, image or an impulse to publicly expose myself	.43	.48	.25
Doubts about my religious faith	.41	.45	.22
An impulse to swear in public	.37	.39	.23
Factor 3: Contamination			
Doubts or images of contamination by touching publicly used door knobs	.74	.68	.70
Doubts or images of contamination after touching garbage or garbage bins	.70	.70	.65
Doubts or images about my hands being dirty after touching money	.64	.58	.65
Doubts or images about being contaminated by germs	.62	.63	.65
Doubts or images of contamination after touching an animal	.61	.57	.63
Doubts or images about catching a disease from public restrooms	.58	.62	.56
Doubts or images about being contaminated, even after slight contact with bodily fluids (sweat, saliva, urine…)	.58	.62	.57
Doubts about being contaminated without knowing it	.48	.52	.49
Factor 4: Aggressive			
Thoughts, images or impulses to hurt defenseless people	.62	.55	.61
Thoughts, images or impulses to push someone (from the bridge, out of the window, into a running traffic…)	.56	.63	.52
Thoughts, images or impulses about attacking someone	.50	.50	.44
Thoughts, images or impulses to hurt animals	.48	.46	.49
Thoughts, images or impulses to drive a car into something or someone	.48	.49	.42
Thoughts, images or impulses of harming myself or others	.47	.48	.40

Thoughts, images or impulses involving weapons or sharp objects	.47	.55	.45
Sexually unwanted thoughts, images or impulses involving defenseless people	.46	.50	.44
Thoughts, images or impulses to steal something	.36	.25	.42
Factor 5: Illness fear			
Doubts about harming others by spreading germs	.51	.59	.44
Doubts about being poisoned by chemical substances (household cleaning products, poisonous substances, radiation…)	.49	.58	.23
Doubts about catching a fatal disease (AIDS, Ebola …)	.45	.48	.22
Doubts about having an illness of which the existence is not yet known	.31	.33	.12
Factor 6: Religious Doubts			
Doubts about performing a religious task or ritual in the right way	.67	.56	.19
Doubts that I might offend God	.48	.36	.34

Note: "Combined" refers to factor loadings of the pooled data. "R-TR" refers to rotated factor loadings of Turkey and "R-BE" refers to rotated factor loadings of Belgium towards the combined sample solution.

3 Supplementary table

Table 3. Turkish-unique factorial structure

	Bad-self	Contamination	Just-right	Accidental harm	BE-Rotated
Thoughts contradictory to my sexual orientation	**.65**		.26		.37
Push someone (from the bridge …)	**.62**				.49
Blurt out obscenities in public	**.61**			.23	.55
People with whom sex is inappropriate	**.61**		.20		.52
Sexual thoughts involving strangers	**.59**				.52
Publicly expose myself	**.59**				.50
Shout out blasphemous words	**.59**			.22	.42
Violent sexual acts	**.58**				.63
Acting immorally	**.57**		.38		.49
Sexual thoughts involving defenseless people	**.57**				.65
Harming myself or others	**.56**		.35		.48
Hurt defenseless people	**.56**				.64
Thoughts contradictory to my moral values	**.55**		.38		.60
Weapons or sharp objects	**.52**				.50
Inappropriate things in a religious context	**.52**	.20	.20		.29
Involving important religious figures	**.49**	.21			.28
Attacking someone	**.49**			.28	.63
Drive a car into something or someone	**.44**			.25	.38

Doubts about my religious faith	**.43**				.02
Swear in public	**.41**			.40	.28
Steal something	**.34**				.52
Hurt animals	**.33**				.50
Contaminated by garbage or garbage bins		**.72**			.68
Contaminated by germs		**.71**		.23	.77
Contaminated by publicly used door knobs		**.67**			.73
Contaminated by bodily fluids		**.64**	.21		.59
Catching disease from public restrooms		**.64**			.57
Contaminated by touching an animal		**.62**	.21		.66
Getting dirty by touching money		**.60**			.64
Contaminated without knowing it	.25	**.56**	.23		.57
Poisoned by chemical substances		**.49**		.35	.30
Catching a fatal disease (AIDS, Ebola …)		**.42**		.41	.46
Leaving a door or window unlocked		**.40**		.28	.22
Harming others by spreading germs	.21	**.39**		.31	.44
Objects might be arranged in a wrong way		**.38**	.22	.25	.14
Having an illness not yet known	.23	**.36**		.28	.33
Accidents involving a loved one		**.32**	.22	.20	.21
Whether I expressed myself in the right way		.21	**.65**	.20	.53
My words or acts will be interpreted as hurtful			**.64**		.43
Whether my work is still being incomplete	.23		**.60**		.54
Performing a religious task in the right way	.24	.27	**.59**	-.21	-.07
Whether I actually carried things out	.26		**.57**	.24	.53
Whether I did things in the way…		.27	**.50**		.50
Doubts that I might offend God	.27	.35	**.44**		-.05
Skipping important information…		.29	**.43**		.26
My forgetfulness put people at risk...		.22	**.34**	.28	.48
Causing disastrous consequences to loved ones	.20	.33	.34	**.50**	.30
Whether I switched off the lights, stove or iron		.35	.29	**.44**	.43
Accidentally hitting a pedestrian while driving		.21	.28	**.40**	.27
Accidentally causing harm to other people…	.22	.20	.35	**.35**	.40

4 Supplementary table

Table 4. Four-factor Turkish solution: Internal-consistency coefficients and Tucker Phi values.

	Bad-self (22 items)	Contamination (15 items)	Just-right (9 items)	Accidental harm (4 items)
Cronbach-Alpha	0.90	0.88	0.84	0.72
Tucker Phi	0.93	0.94	0.82	0.83

Note. Tucker Phi values show the factorial fit of the Belgian loadings to the Turkish structure.

5 Supplementary table

Table 5. Belgian unique factorial structure

	Contamination	Doubts-mixed	Aggressive	Sexual	Hurt-doubts	Religious	TR-R
Contaminated by germs	**.76**						.68
Contaminated by door knobs	**.71**	.20					.64
Contaminated by garbage...	**.67**	.20					.69
Contaminated by touching an animal	**.64**						.61
Getting dirty by touching money	**.63**						.58
Contaminated by bodily fluids	**.59**						.61
Catching disease public restrooms	**.57**						.58
Contaminated without knowing it	**.56**	.24					.54
Catching a fatal disease	**.47**				.29		.40
Harming others by spreading germs	**.43**		.27		.22		.44
Poisoned by chemical substances	**.30**						.50
Whether I switched off the lights...		**.66**					.53
Whether I actually carried things out		**.66**					.43
Leaving a door or window unlocked		**.60**					.35
Whether my work is ... incomplete		**.57**			.23		.39
Whether I did things …	.22	**.54**					.46
My forgetfulness put people at risk		**.53**					.32
Whether I expressed myself…		**.51**			.32		.50
Objects might be arranged…		**.39**		.22			.41
Accidents involving a loved one		**.37**			.23		.36

Accidentally hitting a pedestrian...		**.30**	.21		.26		.45
Skipping important information...		**.30**					.31
Hurt defenseless people			**.80**	.23			.57
Blurt out obscenities in public			**.66**			.45	.52
Hurt animals			**.64**				.35
Publicly expose myself			**.58**				.40
Attacking someone			**.48**	.37	.21		.40
Acting immorally			**.41**	.25			.31
Push someone (from the bridge …)			**.39**	.35	.20		.45
Drive a car into something...			**.34**	.23	.24		.42
Swear in public			**.25**			.23	.47
Sexual thoughts involving strangers				**.68**			.48
Sexual thoughts defenseless people				**.66**			.45
Thoughts contradictory to my values				**.63**		.32	.49
Violent sexual acts			.25	**.62**			.46
People with whom sex is …				**.61**			.40
Thoughts my sexual orientation		.25		**.51**			.43
Steal something			.34	**.39**			.23
Weapons or sharp objects			.32	**.37**	.34		.52
Inappropriate things in a religious…				**.26**			.44
My words … interpreted as hurtful		.43			**.52**		.53
Causing harm without knowing it		.38			**.41**		.35
Having an illness not yet known	.34				**.39**		.19
Harming myself or others			.29	.35	**.37**		.39
Causing disastrous ... to loved ones	.20	.27		.26	**.34**		.23
Thoughts about religious figures			.28		**-.29**		.06
Shout out blasphemous words			.43			**.53**	.21
Doubts that I might offend God						**.46**	.09
Performing a religious task…						**.44**	.18
Doubts about my religious faith						**.30**	.28

6 Supplementary table

Table 6. Six-factor Belgian solution: Internal-consistency coefficients and Tucker Phi values.

	Contamination (11 items)	Doubts-mixed (11 items)	Aggressive (9 items)	Sexual (9 items)	Hurt-doubts (5 items)	Religious (4 items)
Cronbach Alpha	0.86	0.82	0.74	0.82	0.71	0.59
Tucker Phi	0.92	0.94	0.88	0.73	0.74	0.35

Note. Tucker Phi values show the factorial fit of the Turkish loadings to the Belgian structure.
